# Navigated versus non‐navigated total knee arthroplasty: A large single‐implant cohort analysis of clinical outcomes and survivorship

**DOI:** 10.1002/jeo2.70408

**Published:** 2025-09-04

**Authors:** Alessandro Carrozzo, Régis Pailhé, Ophélie Manchec, Sebastien Lustig, Émilie Bérard, Etienne Cavaignac

**Affiliations:** ^1^ Department of Orthopaedic Surgery Hôpital Pierre Paul Riquet, CHU de Toulouse Toulouse France; ^2^ Dipartimento di Scienze della Vita, della Salute e delle Professioni Sanitarie Università degli Studi “Link Campus University” Rome Italy; ^3^ Clinique Aguilera Ramsay Santé Biarritz Biarritz France; ^4^ Orthopaedics Surgery and Sports Medicine Department Lyon University Hospital, FIFA Medical Centre of Excellence, Croix‐Rousse Hospital Lyon France; ^5^ Department of Clinical Epidemiology and Public Health CERPOP, INSERM‐University of Toulouse III, Toulouse University Hospital (CHU) Toulouse France

**Keywords:** clinical outcomes, complications, implant survival, navigation, total knee arthroplasty

## Abstract

**Purpose:**

The aim of this study was to compare implant survival, clinical outcomes and radiographic alignment between navigated and non‐navigated total knee arthroplasty (TKA) performed with a single implant system.

**Methods:**

A retrospective multicenter analysis of prospectively collected data from 6078 TKAs performed for primary osteoarthritis using a single implant system was performed. Procedures were divided into two groups: navigated (*n* = 3602) and non‐navigated (*n* = 2476). The primary outcome was implant survival. Secondary outcomes included re‐intervention rates, patient‐reported outcomes measures (PROMs, International Knee Society scores), and range of motion.

**Results:**

Five‐year implant survival was similar between the non‐navigated (98.9%; 95% confidence interval [CI], 98.2%–99.3%) and navigated (98.3%; 95% CI, 97.6%–98.8%) groups (*p* = 0.505). After adjustment for potential confounders, navigated procedures showed a slightly higher risk of surgical reintervention excluding infection (hazard ratio 1.42, 95% CI: 1.02–1.96, *p* = 0.036). PROM analyses were restricted to patients with both baseline and 5‐year questionnaires (*n* = 470). The improvement of patient‐reported functional outcomes at 5 years was not significantly different between groups (*p* = 0.893 after adjustment for potential confounders).

**Conclusions:**

Navigation was equivalent to conventional instrumentation TKA with respect to implant survival. After adjusting for confounders and excluding septic revisions, navigated procedures showed a slightly higher reoperation risk. No significant advantage in functional outcomes was observed at mid‐term follow‐up. These findings do not support a clear clinical benefit for routine use of navigation in this setting.

**Level of Evidence:**

Level III, retrospective comparative study.

**Abbreviations**
ASA: American Society of Anesthesiologists classification
BMI: body mass index
CAS: computer‐assisted surgery
CI: confidence interval
CNIL: Commission Nationale de l'Informatique et des Libertés (French Data Protection Authority)
IKS: International Knee Society Score
IQR: interquartile range
PROMs: patient‐reported outcome measures
SD: standard deviation
TKA: total knee arthroplasty

## INTRODUCTION

Total knee arthroplasty (TKA) is a well‐established procedure for end‐stage knee osteoarthritis that can significantly reduce pain and improve function [12]. However, despite its success, long‐term implant survival remains an important issue and accurate placement of prosthetic components have historically been considered important determinants of implant survival [4, 10]. In an effort to improve component positioning, computer‐assisted surgery (CAS) with navigation has been introduced as a means to guide bony resection and alignment during TKA more reliably than conventional instruments [5, 7, 9].

Numerous studies have shown that navigated TKA can improve accuracy and reduce the proportion of alignment ‘outliers’ compared with conventional techniques [5, 11].

Although some registry data have linked navigated TKA with fewer aseptic loosening events, especially in younger patients, others have found no significant improvement in patient‐reported outcomes or implant survival at mid‐ to long‐term follow‐up [3, 5, 6, 13]. The conflicting nature of these findings emphasises the complexity of the factors that influence TKA longevity, including patient‐specific factors, surgical technique and implant design.

The aim of this study was to compare the survival and clinical outcomes of navigated versus non‐navigated TKA in a large contemporary cohort, utilising a single implant type.

Given the conflicting evidence regarding its clinical benefits, we hypothesised that navigated TKA will demonstrate equivalence in implant survival compared to non‐navigated TKA with similar clinical outcomes when performed using a single implant.

## METHODS

This study was designed as a retrospective, multicenter cohort analysis utilising prospectively collected data from the Amplitude® database. The registry contained information on patients who underwent primary TKA using the SCORE I implant for primary osteoarthritis. The study included data from 15 centres across France, where surgeries were performed by 16 orthopaedic surgeons. The use of the database was conducted under the authorisation of the CNIL, registered in CliniRecord under the N° 1355265. Amplitude® has registered the data for the long‐term evaluation of the SCORE prosthesis on the public platform ‘Health Data Hub’ under number N° F20210913151920. All data used in this study are sourced from this registry, which we managed according to the CNIL standard methodology MR‐004.

All patients who underwent primary total knee arthroplasty between March 2002 and July 2022 were included in the study. Exclusion criteria comprised cases with missing data on the fixation method, patients with inflammatory arthritis (such as rheumatoid arthritis), and those who received reconstructive prostheses, primarily for tumour‐related conditions.

The primary outcome was implant survival, defined as the time from TKA implantation to revision surgery for any cause, including aseptic loosening, infection, or mechanical failure. Patients were censored at the last available follow‐up or at the time of death if no revision had occurred.

In addition to implant survival, secondary outcomes included surgery‐free survival, evolution of patient‐reported outcome measures (PROMs) measured preoperatively and at the 5 years follow‐up as the International Knee Society (IKS) score and evolution of knee range of motion. The final analyses on IKS were performed on patients having both the pre‐operative and at 5 years follow‐up scores.

Specific complications related to navigation were recorded. Follow‐up assessments were conducted at regular intervals, and the median follow‐up was 24 months (inter‐quartile range [IQR]: 12–62).

### Implant characteristics, surgical technique and navigation protocol

#### Implant

All TKAs were performed with the single‐implant SCORE I system (Amplitude, Valence, France). It is a congruent, posterior cruciate ligament sacrificing, mobile‐bearing design with a rotating platform insert. The femoral component has a constant sagittal radius from full extension to 100° of flexion; stability in deeper flexion is provided by a polyethylene spine that engages the cam. The tibial component uses a cylindrical keel with delta wings for rotational stability.

#### Conventional (non‐navigated) technique

A standard medial or lateral parapatellar approach was used. Femoral and tibial resections followed a mechanical‐alignment philosophy with intramedullary femoral and intramedullary or extramedullary tibial guides. Gap balancing and soft‐tissue releases were performed at the surgeon's discretion.

#### Computer‐assisted (navigated) technique

Navigation was performed with the AMPLIVISION® optical system. After placement of tracked arrays, anatomical landmarks (hip centre, knee centre, malleoli and tibial plateau) were digitised to define the mechanical axes. Real‐time feedback guided coronal and sagittal alignment, tibial slope, femoral rotation and flexion–extension gap symmetry. Component rotation was referenced to the surgical epicondylar axis (femur) and Akagi's line (tibia), described as the axis extending from the centre of the PCL to the medial edge of the patellar tendon [1]. Final component positions were recorded by the software and stored in the registry.

### Statistical analysis

All analyses were performed using STATA software, version 18.0 (StataCorp, College Station, TX, USA). All reported *p*‐values were two‐sided and the significance threshold was < 0.05.

For the primary endpoint, a sample size of 2476 TKA without navigation and 3602 TKA with navigation achieve 90% power to detect equivalence with a margin of ±2%, a reference group proportion expected to 98% and an actual difference fixed to 1%. The significance level is 0.05.

Before analyses, verification of missing or aberrant or inconsistent data was conducted. After corrections, the database was locked. Analysis was performed on the locked database.

Characteristics of patients were first described using the appropriate descriptive statistics according to the type of variables. Descriptive statistics included mean with standard deviation (SD) and median with IQR, for continuous variables, and number of non‐missing observation with frequency (%) for categorical variables. Then, categorical variables were compared between groups using the *χ* ^2^‐test (or Fisher's exact test when necessary). Student's t‐test was used to compare the distribution of continuous variables (or Mann–Whitney's test when distribution departed from normality or when homoscedasticity is rejected).

For the analysis of the survival endpoints, Kaplan–Meier survival curves were described together with 95% confidence interval (CI) and compared, first using the log‐rank test, and then using a cox model adjusted for age, sex, body mass index (BMI) and period (2002–2011 vs. 2012–2022). Finally, as a sensitivity analysis, the propensity score method was used to more extensively take into account potential baseline differences between TKA with and without navigation. A multivariate logistic regression model was generated to estimate for each patient a propensity score to receive navigation. Covariates were period of intervention, age, sex, BMI, ASA, approach, tourniquet, cementation, patellar procedure and patellar type. The performance of the model was appreciated with the c‐statistic (0.75 (95% CI: 0.74–0.77]). Mean propensity score was 0.671 ( ± 0.176) in patients with navigation (*N* = 3602) and 0.479 (±0.210) in non‐navigated group (*N* = 2476). According to propensity score, 1712 patients with navigation were matched with 1712 non‐navigated group (2236 with a precision of 0.0001, 134 with a precision of 0.001, 520 with a precision of 0.01 and 534 with a precision of 0.1). Mean propensity score was the same in patients with and without navigation in the matched sample (0.568 ± 0.184). Survival rate before revision, surgery‐free survival rate and surgery‐free survival rate without sepsis were compared between patients with and without navigation in the subgroup of propensity score matched subjects.

Student's t‐test (or Mann–Whitney's test if necessary) was used to compare the distribution of continuous secondary endpoints. The evolution of IKS and flexion at 5 year was also compared between groups after adjustment for preoperative value, age, sex, BMI and period, using a linear regression model.

## RESULTS

### Patient population and demographics

**Figure 1:**
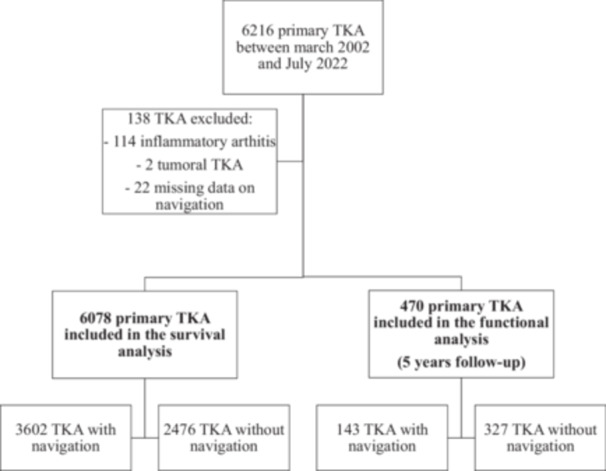
The study flowchart in line with the STROBE (Strengthening the Reporting of Observational Studies in Epidemiology) statement (http://www.strobe-statement.org). TKA, total knee arthroplasty.

A total of 6078 primary TKAs were included in this primary analysis, including 2476 TKA without CAS navigation ('non‐navigated' group) and 3602 TKA with CAS navigation ('navigated' group). A flow chart illustrating the inclusion of patients and the distribution between non‐navigated and navigated TKA is presented in Figure 1.

Demographics of the included patients and intraoperative data are represented in Table 1.

**Table 1:** Patients demographics and intraoperative characteristics.

	Non‐navigated (*n* = 2476)	Navigated (*n* = 3602)	*p*‐Value
Age (years)			0.709
Mean (SD)	71.57 (9.16)	71.48 (8.77)	
Median [IQR]	72 [65–78]	73 [66–78]	
Sex, *n* (%)			0.219
Male	947 (38.8)	1296 (37.2)	
Female	1495 (61.2)	2187 (62.8)	
BMI (kg/m²)			0.050
Mean (SD)	29.88 (5.45)	30.16 (5.25)	
Median [IQR]	29 [26–33]	29 [26–33]	
ASA classification, *n* (%)			<0.0001
1	291 (13.9)	283 (8.7)	
2	1200 (57.4)	2175 (66.5)	
3	595 (28.5)	793 (24.3)	
4	3 (0.1)	18 (0.6)	
5	2 (0.1)	1 (0.0)	
Period of inclusion, *n* (%)			<0.0001
2002–2006	484 (19.5)	640 (17.8)	
2007–2011	499 (20.2)	1612 (44.8)	
2012–2016	1092 (44.1)	1197 (33.2)	
2017–2022	401 (16.2)	153 (4.2)	
Femorotibial components fixation, *n* (%)			<0.0001
Cementless	2012 (81.3)	2537 (70.4)	
Cemented	241 (9.7)	476 (13.2)	
Only tibia cemented	157 (6.3)	278 (7.7)	
Only femur cemented	66 (2.7)	311 (8.6)	
Surgical approach, *n* (%)			<0.0001
Medial parapatellar	2179 (94.9)	2951 (90.1)	
Lateral	116 (5.1)	323 (9.9)	
Tourniquet use, *n* (%)			<0.0001
Yes	180 (8.8)	93 (4.0)	
No	1867 (91.2)	2239 (96.0)	
Patellar procedure, *n* (%)			<0.0001
Yes	1398 (56.5)	1265 (35.1)	
No	1078 (43.5)	2337 (64.9)	
Patellar type, *n* (%)			<0.0001
Non‐cemented inset patella	259 (18.5)	288 (22.8)	
Cemented inset patella	429 (30.7)	179 (14.2)	
Cemented resurfaced patella	710 (50.8)	798 (63.1)	

Abbreviations: ASA, American Society of Anesthesiologists; BMI, mody mass index; IQR, interquartile range; SD, standard deviation.

The groups had a similar age and gender distribution, with a mean age of 71.5 years and approximately 38% men overall. BMI was slightly higher in the navigated group (mean 30.2 vs. 29.9 kg/m², *p* = 0.050). Patients undergoing navigated TKA had significantly fewer American Society of Anesthesiologists (ASA) 1 classifications and higher rates of ASA 2 status compared to the non‐navigated group (*p* < 0.0001). The majority of patients were enroled in the navigated group before 2011 and in the non‐navigated group after 2011 (*p* < 0.0001).

Preoperative IKS scores were slightly higher in the navigated group (mean 98.8 vs. 93.1, *p* < 0.0001) (Table 2).

**Table 2:** Comparison of surgical reintervention causes stratified by group (non‐navigated vs. navigated).

	Non‐navigated (*n* = 2476)	Navigated (*n* = 3602)	*p*‐Value
			0.114
Surgical reintervention for any cause, *n* (%)			
No	2395 (96.7)	3456 (95.9)	
Yes	81 (3.3)	146 (4.1)	
Cause of reintervention, *n* (%)			0.862
Aseptic loosening	5 (6.2)	10 (6.8)	
Patella‐related	10 (12.3)	20 (13.7)	
Infection (sepsis)	23 (28.4)	31 (21.2)	
Fracture	15 (18.5)	29 (19.9)	
Stiffness	23 (28.4)	50 (34.2)	
Pain	4 (4.9)	5 (3.4)	
Dislocation	1 (1.2)	1 (0.7)	

*Note*: Data are presented as *n* (%).

### Primary endpoint: Survival before revision

**Figure 2:**
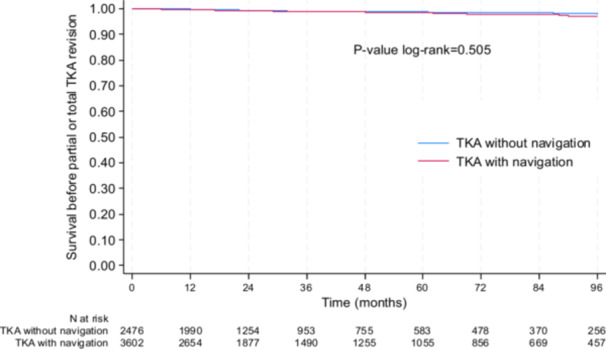
Kaplan–Meier plot demonstrating no differences in TKA survivorship between groups. TKA, total knee arthroplasty.

The overall revision rate was low in both groups (1.0% in the non‐navigated group versus 1.2% in the navigated group; *p* = 0.358). Kaplan–Meier cumulative survivorship of TKAs, stratified by group, is reported in Figure 2.

At 5 years, the survival rate before revision was 98.9% (95% CI: 98.2–99.3) in the non‐navigated group and 98.3% (95% CI: 97.6–98.8) in the navigated group (*p* = 0.505). The equivalence margin of ±2% was not reached.

Using a Cox model adjusted for age, sex, BMI and period of procedure, there was still no significant difference in the risk of revision between the navigated and non‐navigated groups (*p* = 0.527). No significant interactions were found between navigation and age, sex or BMI, suggesting that the effect of navigation did not differ significantly between these subgroups.

The results were the same in the sensitivity analyses conducted in the subgroup of propensity score matched subjects since at 5 years, the survival rate before revision was 99.1% (95%CI: 98.2–99.5) in the non‐navigated group and 98.3% (95%CI: 97.1–99.1) in the navigated group (*p* = 0.264).

### Secondary endpoints

#### Overall reinterventions

**Figure 3:**
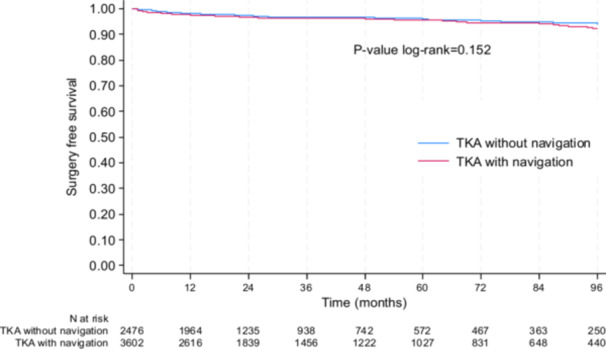
Kaplan–Meier plot for surgery‐free survival.

Surgical reinterventions for any cause (including washout, manipulation under anaesthesia, fracture fixation, or patellar procedures) showed a tendency in favour to the non‐navigated group: at 5 years, the surgery‐free survival rate was 96.1% (95% CI: 94.9–97.0) in non‐navigated group and 95.5% (95% CI: 94.5–97.3) in the navigated group (*p* = 0.152) (Figure 3).

After adjusting for age, sex, BMI and period of surgery, navigated TKAs, there was still a tendency in favour to the non‐navigated group (*p* = 0.071). No significant interactions were found between navigation and age, sex or BMI, suggesting that the effect of navigation did not differ significantly between these subgroups.

The results were also the same in the sensitivity analyses conducted in the subgroup of propensity score matched subjects since at 5 years, the surgery‐free survival rate was 96.1% (95%CI: 94.8–97.2) in the non‐navigated group and 94.6%, (95%CI: 92.9–95.8) in the navigated group (*p* = 0.063).

Rates of septic complications, stiffness, postoperative pain and periprosthetic fractures were similar (*p* = 0.862). Data regarding surgical revision are represented in Table 2.

**Figure 4:**
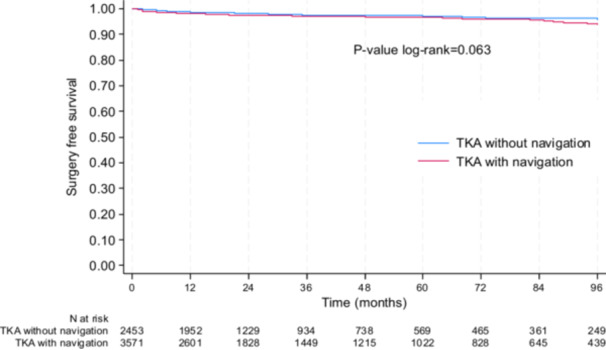
Kaplan–Meier plot for surgery‐free survival (after excluding septic revisions).

After excluding septic revisions (*N* = 54), surgical reinterventions showed a tendency in favour to the non‐navigated group: at 5 years, the surgery‐free survival rate was 97.2% (95% CI: 96.2–98.0) in non‐navigated group and 96.6% (95% CI: 95.8–97.3) in the navigated group (*p* = 0.063) (Figure 4).

The results were the same in the sensitivity analyses conducted in the subgroup of propensity score matched subjects: at 5 years, the surgery‐free survival rate without sepsis was 97.2 (95% CI: 96.0–98.1) in the non‐navigated group and 96.1% (95% CI: 94.7–97.1) in the navigated group (*p* = 0.080). Moreover, after adjusting for age, sex, BMI and period of surgery, navigated TKAs demonstrated a significantly higher risk of all‐cause revision (excluding septic revision) (hazard ratio = 1.42 [95% CI: 1.02–1.96], *p* = 0.036 in the Cox model). No significant interactions were found between navigation and age, sex or BMI, suggesting that the effect of navigation did not differ significantly between these subgroups.

#### Functional outcomes at 5 years

PROM analyses were restricted to patients with both baseline and 5‐year questionnaires (*n* = 470). Comparison of flexion and IKS scores are represented in Table 3. Functional scores (IKS) and range of motion (flexion in degrees) improved from baseline in both groups.

**Table 3:** Comparison of clinical outcomes stratified by group (non‐navigated vs. navigated).

Measure	Non‐navigated	Navigated	*p*‐Value
Preoperative flexion (°)	112.21 (13.44)	111.78 (14.17)	0.239
Flexion at 5 years follow‐up (°)	116.68 (9.38)	118.35 (12.69)	0.158
Evolution of flexion (°)	6.56 (12.88)	5.63 (14.77)	0.530
Preoperative IKS	93.05 (20.91)	98.81 (21.49)	<0.0001
IKS at 5 years follow‐up	182.15 (20.26)	180.98 (21.38)	0.859
Evolution of IKS	90.59 (26.18)	79.80 (26.31)	<0.001

*Note*: Data are presented as mean (standard deviation). Evolution was considered as the postoperative value minus preoperative value.

Abbreviation: IKS, Knee Society Score (0–200).

When evaluating the improvement in IKS from preoperative to 5‐year follow‐up, the non‐navigated group showed significantly greater improvement (*p* < 0.001). However, this difference lost statistical significance after adjustment for preoperative IKS, age, sex, BMI, and intervention period (2002–2011 vs. 2012–2022) (*p* = 0.893). The improvement in range of motion from preoperative to 5‐year follow‐up was not significantly different between groups (*p* = 0.530), even after adjustment for preoperative flexion, age, sex, BMI and period (*p* = 0.081).

#### Specific complications related to navigation

Specific complications related to the navigation system occurred in 0.8% of the patients. The majority of complications occurred at the interface between the bone and the navigation trackers, particularly broken or loosened pins and displaced sensors. True software or hardware failures were less common, but typically resulted in an immediate switch to conventional instrumentation. Metal fragments were retained in a small number of cases, with one patient requiring early re‐operation. No vascular or neurological complications were reported. Specific complication related to navigation are reported in Table 4.

**Table 4:** Specific complications related to navigation.

Event category	No. of events	Consequences
Fixation‐pin problems	10	Abandonment of navigation Two cases left a fragment in the femur/tibia (one needed a second operation to extract a 4‐cm piece)
Tracker instability	9	Loss of accuracy leading to conversion to conventional technique
Navigation system failure	5	Intra‐operative switch to traditional instrumentation
Hardware conflict with trial implants or cutting blocks	4	Navigation interruption, no final acquisition

## DISCUSSION

The primary finding was that navigated TKA showed equivalent implant survival compared to non‐navigated TKA. Specifically, at 5 years of follow‐up, implant survival rates were 98.9% for non‐navigated versus 98.3% for navigated (*p* = 0.505), consistent with our initial hypothesis.

Our results are in line with those of different previous studies. Lee et al conducted a retrospective study with over 11 years of follow‐up compared navigation‐assisted and conventional TKA in patients with severe preoperative varus deformity (HKA > 15°). Although navigation significantly reduced the proportion of HKA alignment outliers (8.1% vs. 18.4%; *p* = 0.04), long‐term clinical outcomes and implant survivorship were similar between groups, with both showing high survival rates (>96%) [6].

The Australian Orthopaedic Association National Joint Replacement Registry previously reported a modest benefit of navigation on implant survival, particularly in younger patients, and attributed this benefit to better component positioning and alignment [3, 13]. In our cohort the implant survival rate was similar in the two groups (*p* = 0.505). However, after adjusting for demographic and surgical confounders, navigated TKAs showed a slightly increased risk of all‐cause surgical reintervention (excluding sepsis), with a hazard ratio of 1.42 (95% CI: 1.02–1.96; *p* = 0.036). The clinical relevance of this statistical finding must be interpreted with caution, especially given the low absolute revision surgeries rates observed.

A substantial body of literature reports improved coronal alignment with navigated TKA [6, 7, 11, 13]. However, although navigation improves radiographic alignment accuracy, this does not necessarily translate into improved functional outcomes or survivorship [7].

In terms of other secondary outcomes, navigated TKA did not significantly outperform non‐navigated TKA in terms of postoperative knee function, with comparable final IKS scores and flexion values at 5‐year follow‐up. Interestingly, the non‐navigated group showed a significantly greater improvement in IKS scores from preoperative baseline. However, this difference lost statistical significance after adjustment for confounding variables. Also, the significantly higher pre‐operative IKS scores in the navigated group may have limited the potential for measurable functional improvement, creating a ceiling effect and reducing the observed post‐operative gains compared to the non‐navigated cohort.

In the present study, the majority of navigation‐related complications resulted at the interface between the bone and the tracking system, particularly due to broken or loosened pins and displaced sensors. These problems were predominantly technical and resulted in navigation interruption rather than direct patient harm. Still, one patient undergo an early reintervention to remove a broken and mobilised pin. Similarly, in a retrospective analysis of 878 primary TKAs, navigation‐related complications were observed in 2.3% of cases, mainly due to pin loosening (1.2%) and hardware or software failure (1.0%), requiring conversion to conventional techniques. Importantly, no fractures, infections or nerve injuries were reported at the pin sites and conversion did not negatively affect short‐term (2‐year) implant survival [8]. However, the use of navigation does not appear to be associated with a significant increase in complications compared to conventional TKAs. In fact, registry data from the American College of Surgeons National Surgical Quality Improvement Program, which analysed more than 108,000 TKAs between 2010 and 2014, showed that computer‐assisted navigation, used in 3.3% of procedures, was not associated with increased operative time and showed comparable rates of short‐term complications, readmissions, reoperations, and length of hospital stay compared with conventional TKA [2].

### Limitations

This study has several limitations. First, its retrospective design introduces potential selection bias. Second, differences in the timing of inclusion (predominantly navigated cases before 2011, non‐navigated cases thereafter) could introduce confounding related to evolving surgical practices or patient selection criteria. However, our analysis included adjustment for relevant confounders together with the propensity score matching method, and the single‐implant cohort design minimised implant‐related variability. Third, the study combined data from multiple surgeons and institutions, which may have contributed to slight variability in both surgical technique and postoperative management protocols. While all the surgeons were experienced, there isn't a 'surgeon‐level' data in our registry. As individual revision thresholds and surgical volume may differ across surgeons, we cannot exclude that unmeasured variability between surgeons may have influenced the observed revision rates. Fourth, surgeons may have decided whether to use navigation based on patient characteristics, intraoperative assessments, or personal preference; however, this was addressed as far as possible by adjusting analyses and with propensity score matching. Another important limitation is the potential for loss to follow‐up, which may affect the reliability of survival estimates. Also, we had a relatively low rate of revision events in both cohorts, limiting the power to detect small differences in implant survival. Finally, the imbalance in sample size between the navigated and non‐navigated groups in the patients receiving IKS scores comparisons introduces the potential for selection bias. This discrepancy is primarily due to the fact that only a subset of patients had complete preoperative and 5‐year PROM data available, and PROM collection was more consistently implemented by early adopters of navigation. Consequently, group allocation for the functional analysis was not randomised but driven by data availability. So, an adjustment relevant baseline covariates and propensity‐score analyses were conducted to mitigate confounding.

Despite its limitations, this study has several strengths. It represents a large single‐implant cohort of 6078 TKAs, ensuring uniformity in implant‐related factors. The multicenter design, involving 15 centres and multiple surgeons, enhances the external validity of the results and minimises centre‐specific biases. Adjustment for potential confounders, through multivariable analyses and propensity score matching, strengthens the reliability of comparisons. Finally, the use of contemporary surgical techniques and a follow‐up of 5 years allows a valid assessment of mid‐term implant survival and clinical outcomes.

## CONCLUSIONS

In conclusion, our results suggest that navigation is equivalent to over conventional technique for TKA in terms of implant survival. After adjusting for confounding factors and excluding septic revisions, navigated procedures had a slightly higher risk of reoperation, although the absolute difference in reoperation rates was small. Furthermore, at mid‐term follow‐up, none significantly superior functional outcomes were showed with the use of navigation.

These results suggest that the routine use of computer‐assisted navigation TKA does not appear to offer superior implant survival or significantly better clinical outcomes compared to conventional instrumentation when using a single implant system. However, further high‐quality comparative studies with randomisation are warranted to confirm these findings.

## AUTHOR CONTRIBUTIONS

All authors contributed to the study. Etienne Cavaignac and Régis Pailhé have ideated the study and established the study design. Material preparation, data collection and analysis were performed by Etienne Cavaignac, Ophélie Manchec, Régis Pailhé, and Émilie Bérard. The first draft of the manuscript was written by Alessandro Carrozzo, and Etienne Cavaignac, Sebastien Lustig and Ophélie Manchec had substantially edited the draft. All authors read and approved the final manuscript.

## CONFLICT OF INTEREST STATEMENT

Etienne Cavaignac: Consultant for Arthrex, Amplitude and Biobank. Sébastien Lustig: Royalties from Stryker and Smith & Nephew and institutional support from Amplitude. The remaining authors declare no conflicts of interest.

## ETHICS STATEMENT

The use of the database was conducted under the authorisation of the CNIL, registered in CliniRecord under the No. 1355265. Amplitude® has registered the data for the long‐term evaluation of the SCORE prosthesis on the public platform ‘Health Data Hub’ under number No. F20210913151920. All data used in this study are sourced from this registry, which we managed according to the CNIL standard methodology MR‐004.

## Data Availability

The data sets generated and analysed during the current study are not publicly available due to confidentiality agreements but are available from the corresponding author on reasonable request.
